# 

**Published:** 1869-02-01

**Authors:** 


### *CONTENTS.*

ORIGINAL COMMUNICATIONS:

Mental Influence in Physical Disease. An Inaugural Thesis for the Degree of M. D. By John Williamson, Chicago........ 65

Case of Placenta Præmatura. By Ho. Berchelmann, M. D., Belleville, St. Clair County, Illinois........ 84

De-Nicotinized Tobacco. By T. Williams, M. D., Milwaukee........ 87

TRANSLATIONS:

Applied Chemistry. Application of the Atomic or Thermic Law to Biatomic Metalloids. By Dr. Rabuteau. From the Proceedings of the Biological Society, Paris. Report of *Gazette Medicale.* Translated for THE JOURNAL, by W. Hay, M. D., Associate Editor........ 89

Conclusion of Unpublished Investigations. By Dr. Rabuteau........ 92

Investigations into the Semen of Old Men. By A. Dieu, Physician a l'Hotel des Invalides........ 94

EDITORIAL:

Delay........ 94

Rush Medical College Commencement........ 95

